# Reverse Design of On-Chip Terahertz Demultiplexers

**DOI:** 10.3390/mi12091093

**Published:** 2021-09-10

**Authors:** Guofeng Zhu, Feng Huang, Zhenrong Dai, Xuewei Ju, Shuncong Zhong, Xiangfeng Wang

**Affiliations:** School of Mechanical Engineering and Automation, Fuzhou University, Fuzhou 350108, China; N190220063@fzu.edu.cn (G.Z.); 17750414845@163.com (Z.D.); xueweiju1991@gmail.com (X.J.); zhongshuncong@hotmail.com (S.Z.)

**Keywords:** reverse design, terahertz, demultiplexer, FDTD

## Abstract

The reverse design method (RDM) is a frontier direction in the optical research field. In this work, RDM is applied to the design of terahertz demultiplexers, including two-port and three-port terahertz demultiplexers, with areas of 3 mm × 3 mm and 5 mm × 5 mm, respectively. The Finite-Difference Time-Domain (FDTD) simulation results show that the terahertz waves at frequencies of 0.5 THz and 0.417 THz can be well separated by the two-port demultiplexer, and the transmittances of the two outputs reach as high as 0.75 after bandwidth optimization. Meanwhile, the three-port terahertz demultiplexer can have terahertz waves separated from three Ports, and the crosstalk between adjacent channels is less than −18 dB.

## 1. Introduction

Terahertz (THz) technology has broad application prospects in many fields, such as sensing, non-destructive testing, spectroscopy, medical imaging, biology, communication and so on [1,2,3,4]. However, the existing THz systems are composed of many discrete optical components. Compared with modern integrated optical systems, they are bulky and expensive, which is not conducive to facilitate THz applications. Traditional design methods excessively rely on the designer’s experience and optical theories, by continually adjusting the structural parameters of the devices to achieve particular performance, such as THz demultiplexers [5], filters [6,7], polarizers [8] and power splitters [9]. Limited by the theoretical value calculated by the self-imaging analytical theory, it is difficult to make the size of the devices smaller. Miniaturized, integrated and high-performance on-chip THz functional devices are highly desired.

The reverse design method (RDM) as a unique tactic, by treating target performance as assessment standards, utilizing intelligent algorithms to optimize an initial structure and, eventually, finding out a satisfying device. The novel approach, different from traditional methods, accomplish designs by exploring all possible structures in the whole design area, and the final device has more complex functions, a higher performance and a smaller volume [10]. In recent years, RDM has been widely utilized in the design of nanophotonic devices [11], such as a polarization beam splitter designed by a direct binary search algorithm [12], a nanophotonic wavelength router designed by a genetic algorithm [13], an on-chip multi-channel focusing wavelength demultiplexer designed by an objective-first algorithm [14], etc. However, RDM has not been widely applied to the design of the THz devices. A wavelength demultiplexer can effectively expand the wave transmission capacity and improve the integration of the optical system. At present, several methods have been proposed to fabricate demultiplexers, including using ring resonators [15,16], coupled cavity waveguides [17,18], directional coupling [19], a graphene plasmonic cross-shaped resonator [20], metamaterials and metasurfaces [21,22,23]. In this work, silicon-based two-port and three-port on-chip THz demultiplexers are architected by RDM. Finite-Difference Time-Domain (FDTD) simulation results show that the demultiplexers can output THz waves of different frequencies from different Ports, and their transmittances can reach more than 0.75 in a wide frequency range after bandwidth optimization. Low insertion losses and crosstalk can be achieved. RDM can provide solutions for many THz devices which are difficult to be designed by traditional methods and theories.

## 2. Reverse Design Method

Traditional forward design method is generally based on the specific analytical theory. For example, to design a THz demultiplexer based on a photonic crystal structure, firstly, a plane wave expansion method is utilized to calculate its dispersion curve; then, the propagation constant can be extracted from the dispersion curve and be brought into the self-imaging conditions to calculate self-imaging position [24]; finally, the required devices can be designed according to the self-imaging position. The method is not only time-consuming and laborious, but also requires rich personal experience in theoretical analysis. The method will be severely challenged when the applications of the devices are expanded to the broadband, nonlinear phenomena and integrated systems. Especially in the process of nonlinear device optimization, several interdependent characteristic parameters need to be optimized at the same time, for which the personal experience is highly crucial. However, RDM, viewing the functional parameters of the device as the evaluation indexes, utilized intelligent algorithms to find out the optimal structures which satisfy the design requirement. Non-periodic topological device structures can be obtained by searching the entire design space using the algorithms, achieving complex functions and higher performance that could not be realized before.

We constructed a highly efficient RDM based on the objective-first algorithm. The objective-first algorithm first sets up the target electric field distribution, and then optimizes physical residue and generates an optimal structure. The optimization forms are as follows:

(1) $$\mathrm{minimize}\;\sum_{i}^{N}f_{i}\left(x_{i}\right)+g\left(z\right);$$

(2) $$\mathrm{subject}\;\mathrm{to}\;A_{i}\left(z\right)x_{i}-b_{i}\left(z\right)=0,\;for\;i=1\ldots N;$$

where *x_i_* is electric field variable, *z* is the structure variable (dielectric constant in this work), $f_{i}\left(x_{i}\right)$ is the objective function of electric field, g(z) is the structural objective function and $A_{i}\left(z\right)x_{i}-b_{i}\left(z\right)$ in Equation (2) is the physical residue, which is the linear algebra description form of Maxwell’s equations (Equation (3)) in the frequency domain.

(3) $$\nabla \times \mu_{0}{}^{-1}\nabla \times E-\omega^{2}\varepsilon E=-i\omega J$$

where *E* is the electric field intensity, *J* is the excitation current source, ω is the angular frequency, $\mu_{0}$ is the vacuum permeability, ε is the dielectric constant. In order to simplify Equation (3) into Equation (2), we needed to make the following substitutions with algebraic notations: E→x, ε→z, $\nabla \times \mu_{0}^{-1}\nabla \times -\omega^{2}\varepsilon \to A(z)$, −iωJ→b.

In the optimizing process of the objective-first algorithm, firstly, the dielectric constant distribution satisfying the target performance can be calculated using Equation (3) [25]. However, the physical residue will appear in Maxwell’s equations; that is, Equation (2) is not equal to zero. Due to the existence of non-zero physical residue, the final value of electric field will deviate from ideal value. Therefore, an Alternating Direction Method of Multiplier (ADMM) was utilized to alternately optimize the structure and electric field [26] and, when the next structure was confirmed, objective function values could be calculated by FDTD simulation. Iterative optimization was implemented until the physical residue was less than a fixed value or a specific number of iterations was reached. After the iterations were terminated, the algorithm would automatically generate a functional device layout in GDS format that met the target performance.

**Figure 1:**
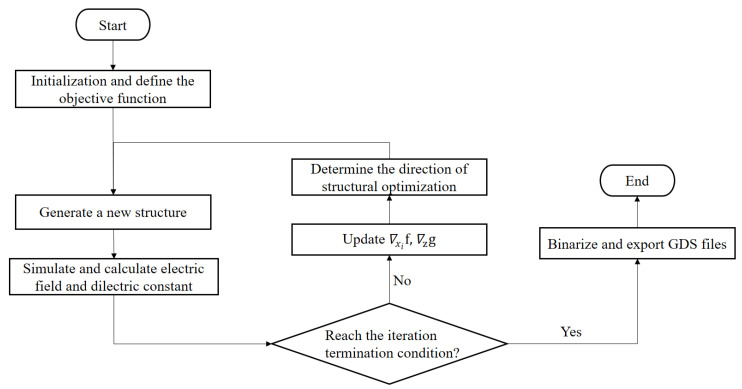
The flow chart of the RDM. $\nabla_{xi}f$ and $\nabla_{z}g$ are the gradients of the objective functions (*f* and *g*) to the electric field (*x_i_*) and dielectric constant (*z*), respectively.

The reverse design process based on the objective-first algorithm and the FDTD method can be divided into four steps: (1) Define the optimization region of the initial structure and the objective function. (2) The objective-first algorithm alternately optimizes the electric field and dielectric constant, and the optimization direction is determined according to the derivatives of the objective functions to the electric field and dielectric constant. When the optimization reaches the termination condition, stop the iteration. (3) Discretize (binarize) the continuously changing dielectric constant to obtain the structure with only silicon and air. (4) Generate the THz demultiplexer structure in GDS format. The flow chart of RDM is shown in Figure 1:

## 3. Results and Discussion

**Figure 2:**
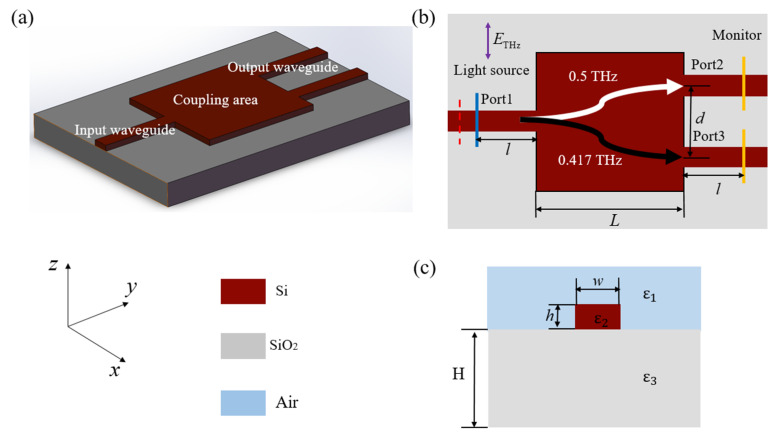
Three-dimensional schematic diagram (**a**), top view (**b**) and waveguide cross section (**c**) of the initial structure for the two-port THz demultiplexer. The blue and orange lines in (**b**) represent the positions of the light source and 2D monitors in the FDTD simulation, respectively. *L* = 3 mm, *d* = 1.5 mm, *w* = 400 μm, *h* = 200 μm, H = 500 μm, ε_1_ = 1, ε_2_ = 11.7 and ε_3_ = 3.8.

As shown in Figure 2a, the initial structure of the two-port THz demultiplexer can be divided into four parts, including an input waveguide, a square coupling area (optimization area), two output waveguides and a substrate. The incident mixing source with frequencies at 0.5 THz and 0.417 THz is TE polarized. The device was designed to separate the two frequencies via the coupling area and output from two output waveguides as shown in Figure 2b. The length *L* of the square coupling area was 3 mm, the distance *d* between the centers of the two output waveguides was 1.5 mm, and the width *w* and height *h* of the input and output Ports were 400 μm and 200 μm, respectively. The device was composed of a high-resistivity silicon (Si) which was placed on a silica substrate (SiO_2_). The dielectric constants of Si and SiO_2_ used in FDTD simulations were 11.70 and 3.80, respectively [27]. The power absorption coefficient of the high-resistivity Si was lower than 0.05 cm^−1^ below 1 THz [27]; thus, we ignored the absorption loss in the simulations. The SiO_2_ substrate only played an insulating and supporting role.

**Figure 3:**
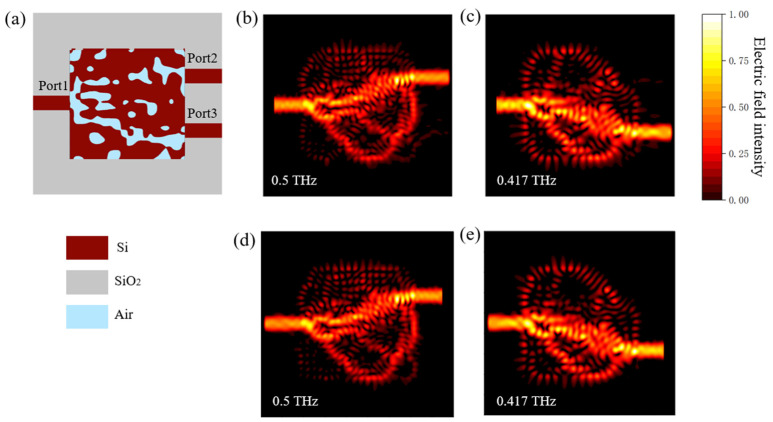
The top view of the final optimized structure diagram for the two-port THz demultiplexer (**a**) and the electric field intensity distribution maps at 0.5 THz (**b**) and 0.417 THz (**c**) when the mixing THz waves were incident from Port 1, at 0.5 THz when the THz wave were incident from Port 2 (**d**) and at 0.417 THz when the THz wave were incident from Port 3 (**e**), indicating that the device also worked as a multiplexer.

After setting initial parameters, we determined the corresponding objective function which defined the device performance, also called the figure of merit (FOM). For demultiplexers, the transmittance at each Port was vital, so the FOM in was set as follows:

(4) $$FOM={\left(1-T_{1}\right)}^{2}+{\left(1-T_{2}\right)}^{2}$$

where T_1_ and T_2_ represent the transmittances at Port 2 (0.5 THz) and Port 3 (0.417 THz), respectively. The smaller the value of FOM is, the higher the transmittances at the different Ports will be. Then, the initial structure and objective function were imported into the reverse design program for optimization, and the final structure was obtained as shown in Figure 3a. The coupling area was composed of two medias, silicon (red area) and air (blue area), where the irregular hollow blue areas could be fabricated by etching the silicon layer on a silica substrate. The target frequencies were 0.5 THz at Port 2 and 0.417 THz at Port 3, respectively. The numerical simulation of the electromagnetic field was carried out by the FDTD method (FDTD Solutions, 2018 version, Lumerical Solutions, Inc., Vancouver, BC, Canada). In the 3D-type FDTD simulations, the mesh size was 20 μm, the input source was a waveguide light source (one option of the light sources in FDTD) which was assumed to be already coupled into the input waveguide with an input power one at each frequency (no unit in FDTD), the number of perfect matching layers (PML) was 10, the boundary condition was the Bloch Boundary and the output monitors were both 2D monitors placed on the plane perpendicular to the output waveguides. These parameters were loaded into the FDTD through the program of the reverse design. After the optimization process at multiple frequency points, we could obtain the final device distributions of the electric field and transmittance spectra at both output ports. The distribution maps of the electric field intensity at 0.5 THz (Port 2) and 0.417 THz (Port 3) are shown in Figure 3b,c. The results clearly showed that a mixing THz wave containing two frequencies (0.5 THz and 0.417 THz) imported from Port 1 was outputted from Port 2 and Port 3 after passing through the coupling region.

When the THz wave was imported from the opposite direction (output Ports), the distributions of electric field intensity remained nearly unchanged, as shown in Figure 3d,e. Therefore, the device designed by RDM worked not only as a demultiplexer, but also as a multiplexer/combiner, which combined THz waves with different frequencies into one port to output.

**Figure 4:**
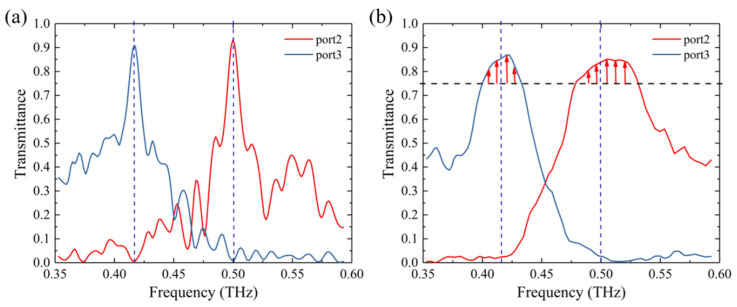
Transmittance curves of the two-port demultiplexer before (**a**) and after (**b**) bandwidth optimization.

We also performed a wide-band (0.355 THz–0.590 THz) simulation for the device, and calculated its transmittance spectra. The results are shown in Figure 4a. At 0.5 THz, the transmittances at Port 2 and Port 3 were about 0.93 and 0.02. Due to the scattering in the coupling area, the total transmittance was less than 1. Similarly, at 0.417 THz, the transmittances at Port 3 and Port 2 were about 0.9 and 0.03. The device showed good performance at the design frequencies, but the transmittance quickly dropped to 0.5 or lower when the frequency was off the central frequency. Fabrication errors such as larger or smaller etched areas always occur during the manufacturing process, resulting in the shift of the operating frequencies deviating from the design frequencies, which could cause a quickly degraded transmittance. Therefore, it is necessary to optimize the bandwidth of the device in order to maintain a high transmittance within a certain frequency range. The objective function for the broadband optimization can be modified as follows:

(5) $$FOM=\sum_{0.484}^{0.516}{\left(1-T_{1}\right)}^{2}+\sum_{0.401}^{0.433}{\left(1-T_{2}\right)}^{2}$$

where T_1_ is the transmittance ranging from 0.484 THz to 0.516 THz and T_2_ is the transmittance ranging from 0.401 THz to 0.433 THz. The frequency step was 0.008 THz. The objective function was brought into the algorithm to optimize the structure at multiple frequency points.

The optimized transmittance curves are shown in Figure 4b. It can be seen that the transmittances decreased at central frequencies, but remained above 0.75 with a range of 0.032 THz around the design frequency. Compared with the THz demultiplexer designed based on photonic crystal structures [19], the demultiplexer based on RDM had a smaller area and was robust in transmittance within a certain frequency range. The crosstalk C*_ij_* between adjacent channels can be calculated as:

(6) $$C_{ij}=10\;\times \;\mathrm{lg}\left(T_{ij}\right)$$

where T*_ij_* is the transmittance of the *i*th Port to the *j*th Port. C_23_ and C_32_ of the two-port demultiplexer were −19 dB and −22 dB after bandwidth optimization, respectively.

**Figure 5:**
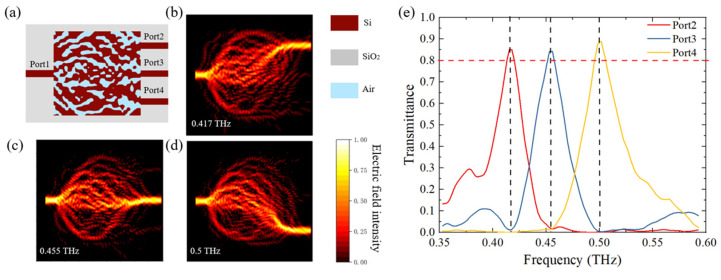
The final optimized structure of the three-port THz demultiplexer (**a**), the distributions of electric field intensity at 0.417 THz, 0.455 THz and 0.5 THz at Port 2-4 (**b**–**d**) and the transmittance curves at these Ports (**e**).

In order to verify the reliability of the RDM, we also designed a three-port demultiplexer with an area of 5 mm × 5 mm, which could separate THz waves with frequencies of 0.417 THz, 0.455 THz and 0.5 THz from three different Ports, respectively. The final optimized structure of the device is shown in Figure 5a. The distance between the centers of two adjacent output waveguides was 1.3 mm. All other parameters were the same as those for the two-port demultiplexer. The distributions of the electric field intensity at these frequencies are shown in Figure 5b–d, and the transmittance curves at these Ports are shown in Figure 5e. The transmittances were above 0.84 at the three output Ports, and the crosstalk between adjacent channels was less than −18 dB.

We summarized the relevant parameters of THz demultiplexers from literatures and our work in Table 1 for comparison. We obtained the transmittance values from the graphs in References [16,18,19,21]. The FWHM in Refs. [16,19,21] and crosstalk in References [16,18,21] were not reported, so we left dashes in Table 1. At present, the design of THz demultiplexers mainly relied on traditional optical methods. The THz demultiplexers by the RDM had a comparable high transmittance and a low crosstalk, an easily optimized bandwidth and a much smaller volume size, which may have practical applications in high-speed THz communications and integrated THz systems. The method can be promoted to design THz switches, couplers, connectors, filters, etc.

In the simulations, THz radiation was assumed to already be injected into the input waveguide. In the real case, THz radiation can be coupled into the waveguide by a tapered channel waveguide, as demonstrated in Reference [28]. The radiation can also be coupled into the waveguide by gluing a silicon columnar lens on the end of the waveguide, or by using a grating coupler as demonstrated in Reference [29]. To couple out the THz radiation, we could use the same couplers as mentioned. One could use silicon-on-glass (SOG) technology as presented in Reference [30] to fabricate the device. The irregular hollow blue areas in Figure 3 and Figure 5 can be fabricated by selective etching the silicon layer on a silica substrate.

## 4. Conclusions

In this work, two-port and three-port on-chip THz demultiplexers were designed using the RDM. The two-port THz demultiplexer could separately output THz waves at 0.417 THz and 0.5 THz from different Ports with the transmittances at the target frequencies above 0.75 within a bandwidth of 0.032 THz and an area of 3 mm × 3 mm. The three-port THz demultiplexer could separately output the THz waves at 0.417 THz, 0.455 THz and 0.5 THz from different Ports with the transmittances at the target frequencies above 0.8 and an area of 5 mm × 5 mm. The channel crosstalks of the devices were lower than −18 dB. The advantages of the devices designed by the RDM lie in the smaller and integrated space, as well as the silicon-based structure, which will promote the development of THz integrated systems in the future and has an important application value for THz technology.

## Figures and Tables

**Table 1:** Comparison of relevant parameters of this work with some others.

Ref.	Methods	Frequencies (THz)	Transmittance	FWHM (GHz)	Crosstalk (dB)
[16]	Ring resonators	6.6, 6.9, 7.6	0.53, 0.36, 0.3	-	-
[18]	Coupled cavity waveguides	1.09, 1.113 1.315	0.76, 0.92 0.8	<2	-
[19]	Directional coupling	0.524, 0.537, 0.560, 0.585	0.91, 0.94 0.93, 0.96	-	−14.45 (average)
[21]	Metamaterial	0.225, 0.410	0.98, 0.97	-	-
This work	RDM	0.417, 0.5	0.86, 0.85	64, 98	−22, −19
		0.417, 0.455, 0.5	0.85, 0.84 0.89	28, 30 39	−18.7, −18.1 −26.4

## Data Availability

Data are contained within the article.
